# Increased stiffness of the tumor microenvironment in colon cancer stimulates cancer associated fibroblast-mediated prometastatic activin A signaling

**DOI:** 10.1038/s41598-019-55687-6

**Published:** 2020-01-09

**Authors:** Jessica Bauer, Md Abdul Bashar Emon, Jonas J. Staudacher, Alexandra L. Thomas, Jasmin Zessner-Spitzenberg, Georgina Mancinelli, Nancy Krett, M. Taher Saif, Barbara Jung

**Affiliations:** 1 Department of Medicine, Division of Gastroenterology and Hepatology, University of Illinois at Chicago, Chicago, IL USA; 2 Department of Mechanical Science and Engineering, University of Illinois at Urbana-Champaign, Urbana, IL USA; 3 Department of Gastroenterology, Infectious Diseases and Rheumatology, Charité-University Medicine, Berlin, Germany; 4 Berlin Institute of Health (BIH), Berlin, Germany; 5 Medical University of Vienna, Vienna, Austria

**Keywords:** Cancer microenvironment, Colon cancer

## Abstract

Colorectal cancer (CRC) is the second deadliest cancer in the US due to its propensity to metastasize. Stromal cells and especially cancer-associated fibroblasts (CAF) play a critical biophysical role in cancer progression, but the precise pro-metastatic mechanisms are not clear. Activin A, a TGF-β family member, is a strong pro-metastatic cytokine in the context of CRC. Here, we assessed the link between biophysical forces and pro-metastatic signaling by testing the hypothesis that CAF-generated mechanical forces lead to activin A release and associated downstream effects. Consistent with our hypothesis, we first determined that stromal activin A secretion increased with increasing substrate stiffness. Then we found that stromally-secreted activin A induced ligand-dependent CRC epithelial cell migration and epithelial to mesenchymal transition (EMT). In addition, serum activin A levels are significantly increased in metastatic (stage IV) CRC patients (1.558 ng/ml versus 0.4179 ng/ml, p < 0.05). We propose that increased tumor microenvironment stiffness leads to stromal cell-mediated TGF-β family signaling relying on the induction and utilization of activin A signaling.

## Introduction

Colorectal cancer (CRC) remains a significant challenge from both public health and clinical perspectives. With approximately 50,000 deaths per year in the US, CRC is the second leading cause of cancer-related mortality in the US^1^. Despite the decreased incidence of CRC over the previous decades, largely due to early detection through enhanced screening, there is now an alarming increase in late-stage CRC in younger patients^2,3^. Five-year mortality of patients with stage IV disease remains as high as 90%^1,4^, and novel approaches are needed for effective risk stratification and treatment.

CRC metastatic potential is strongly influenced by the stroma. Fibroblasts within the stroma, the major cell type composing the stromal cell population^5^, are critical determinants of stromal cross-talk and cancer progression. A subpopulation of these fibroblasts is activated into cancer-associated fibroblasts (CAFs) (also known as myofibroblasts) expressing alpha-smooth muscle actin (α-SMA)^5–9^. Fibroblast can be activated to CAFs by Transforming growth factor β (TGF-β)^7,10^. These CAFs contribute to a dense myofibroblastic component and deposition of extra-cellular matrix (ECM) proteins associate with tumor fibrosis^11^. CAFs generate increasing force leading to increased stiffness on 2D soft substrates *in vitro*^12,13^. *In vivo*, they produce growth factors promoting metastatic progression of cancer cells^14^ and produce collagen and the collagen crosslinker Lysyl oxidase (LOX), which stiffen the extra-cellular matrix (ECM) and remodel its composition and architecture^15^. CAF contractile force can further contribute to changes in tumor biomechanical properties by mechanical non-linear stress-strain deformation leading to compressive stress in the tumor and pressure gradients on the proliferating epithelial cancer cells^16,17^. Additional stress is generated by tumor cell growth in a confined space^16^. Therefore, stromal cells and CAFs are critical biophysical players in cancer progression. Stroma mechanics are particularly relevant for colorectal and pancreatic cancer.

The stiffness of colon tumor ECM increases with cancer progression^15,18^. In turn, CAFs may react to the stiffening microenvironment by generating more force. Latent TGF-β^19,20^ released by the cancer cells is stored in the ECM by binding proteins^21^. Rifkin and associates have determined that ECM mechanical forces activate latent TGF-β^22^ promoting epithelial to mesenchymal transition (EMT) of colon tumors^23,24^. The mesenchymal-like cancer cells have enhanced mobility which allows escape from the cancer cell clusters and leads to migration to distant sites. Our previous work demonstrated that substrate stiffness alone can induce an EMT-like phenotype in colon cancer cell lines^25–27^. Here, we demonstrate a connection between tumor microenvironment (TME) stiffness, CAF-secreted factors, and epithelial cells during metastasis with a focus on TGF-β/activin A pathway signaling.

While TGF-β is well recognized as being important in CAF transformation^7^, its family member activin A has also been implicated in the activation of fibroblasts in the tumor stroma and in the downstream metastatic processes of several solid tumors^28–30^. Downstream of activin receptors, signaling diverges into a canonical SMAD-dependent pathway and SMAD-independent non-canonical pathways^31^. The activin A ligand, various receptors, and crosstalk between canonical and non-canonical downstream signaling create a multi-functional and context-specific signaling network, with a growth-suppressive net effect at early stages of cancer but a pro-metastatic effect with disease progression^31,32^. Using *in vitro* models of CRC, we previously reported that restoration of the frequently mutated activin type II receptor ACVR2A leads to a more metastatic phenotype^28^. Furthermore, we characterized the interplay between activin A and the cell cycle inhibitor p21 and established that activin’s pro-metastatic, non-canonical signaling preferentially utilizes PI3K/AKT signaling while metastatic actions of TGF-β rely on MEK/ERK downstream signaling^28^. Others have shown that CAFs secret activin, which in turn remodels the TME and increases metastatic potential of tumors^33^.

We previously published that TGF-β-induced pro-metastatic phenotype acts via activin A signaling in CRC^30^. Baseline levels of secreted activin A in CRC stromal cells is increased by 10-fold after TGF-β treatment^30^ and further increased by co-culture of stromal with CRC epithelial cells suggesting that the stroma is a significant source of secreted activin A^30^. Given the importance of epithelial-stromal interaction in tumorigenesis and metastasis and recent acknowledgement of not only TGF-β’s but also activin A’s role in stromal effects in CRC^30,34–36^, we assessed ligand-dependent migration in the presence of stromal cells. We showed that TGF-β-induced migration in epithelial cells is significantly higher when fibroblasts are present in a co-culture and is dependent on activin A since specific inhibition of activin with follistatin decreased TGF-β-induced migration. Here we examine the role of tumor stiffness in this process.

Previous studies have established that ECM-bound latent TGF-β is activated by increased stromal stiffness leading to increased EMT^24^. We posit that activin A is a critical intermediate in this signaling pathway. Carracedo *et al*.^37^ observed that fibroblastic integrin α11 induction by increased matrix stiffness leading to myofibroblast differentiation required activin A. Similarly, breast cancer cells treated with TGF-β and cultured on polyacrylamide gels with increasing stiffness demonstrated increased EMT through PI3K/Akt signaling^38^, which suggests the involvement of activin A^28^. We observed increased activin A-mediated prometastatic effects with increasing matrix stiffness, leading us to hypothesize that in response to increasing TME stiffness, CAFs release activin A, which promotes a metastatic tumor environment. In this study, we demonstrate the mechanistic link between stiffness, activin A, and EMT in CRC.

## Results

### Activin A secretion from fibroblasts increases with higher substrate stiffness and induces epithelial cell migration

CAFs play an important role in cancer progression and generate force in the TME^14^. Our recent data suggest that TGF-β-induced pro-metastatic epithelial changes are dependent on activin A secretion in the tumor stroma^30^. However, it is unknown if tumor stiffness contributes to TGF-β /activin A signaling to promote metastasis. We hypothesize that increased microenvironment stiffness promotes CAFs to secret soluble pro-metastatic factors. To test this hypothesis, we measured activin A secretion from CAFs activated by TGF-β and subsequently cultured on substrates of increasing stiffness to measure the downstream impact on tumor epithelial cells.

**Figure 1:**
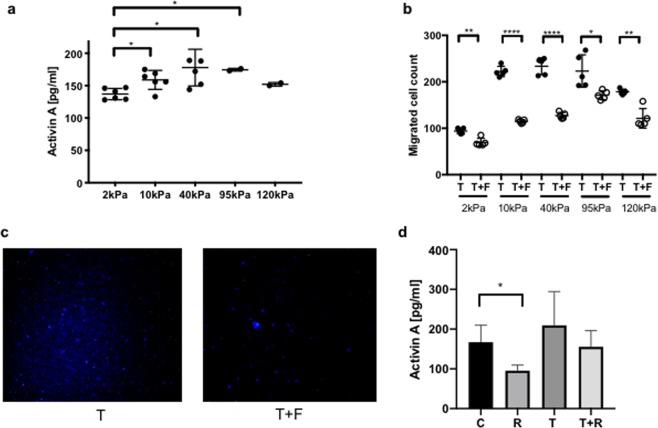
Activin A secretion from fibroblasts increases with higher substrate stiffness and induces epithelial cell migration. (**a**) ELISA measuring secreted activin A from CCD18 fibroblast cells cultured on substrates with increasing stiffness. (**b**) Migration of FET epithelial cells through a transwell following treatment with media collected from CCD18 cells cultured on substrates with increasing stiffness. To demonstrate the specific impact of activin A on cell migration, cells were treated with follistatin (F), an inhibitor of activin A, 30 minutes prior to sample collection. (**c**) Representative pictures of DAPI stained cells of migrated FET from (**b**) treated with TGF-β (T) on the left panel and follistatin (F) 30 min prior TGF-β (T) treatment on the right panel. (**d**) ELISA measuring activin A level secreted from CCD18 fibroblasts cultured on 10kPa substrate. To demonstrate the influence of cell attachment on activin secretion, we treated CCD18 with RGDS peptide (R) prior to TGF-β (T) treatment to disrupt cell attachment via integrins. For Fig. 1, all the cells were treated with TGF-β (T) for 72 hours to induce CAF phenotype and lead to activin A secretion. (*p < 0.05, **p < 0.01, ***p < 0.001, ****p < 0.0001).

**Figure 2:**
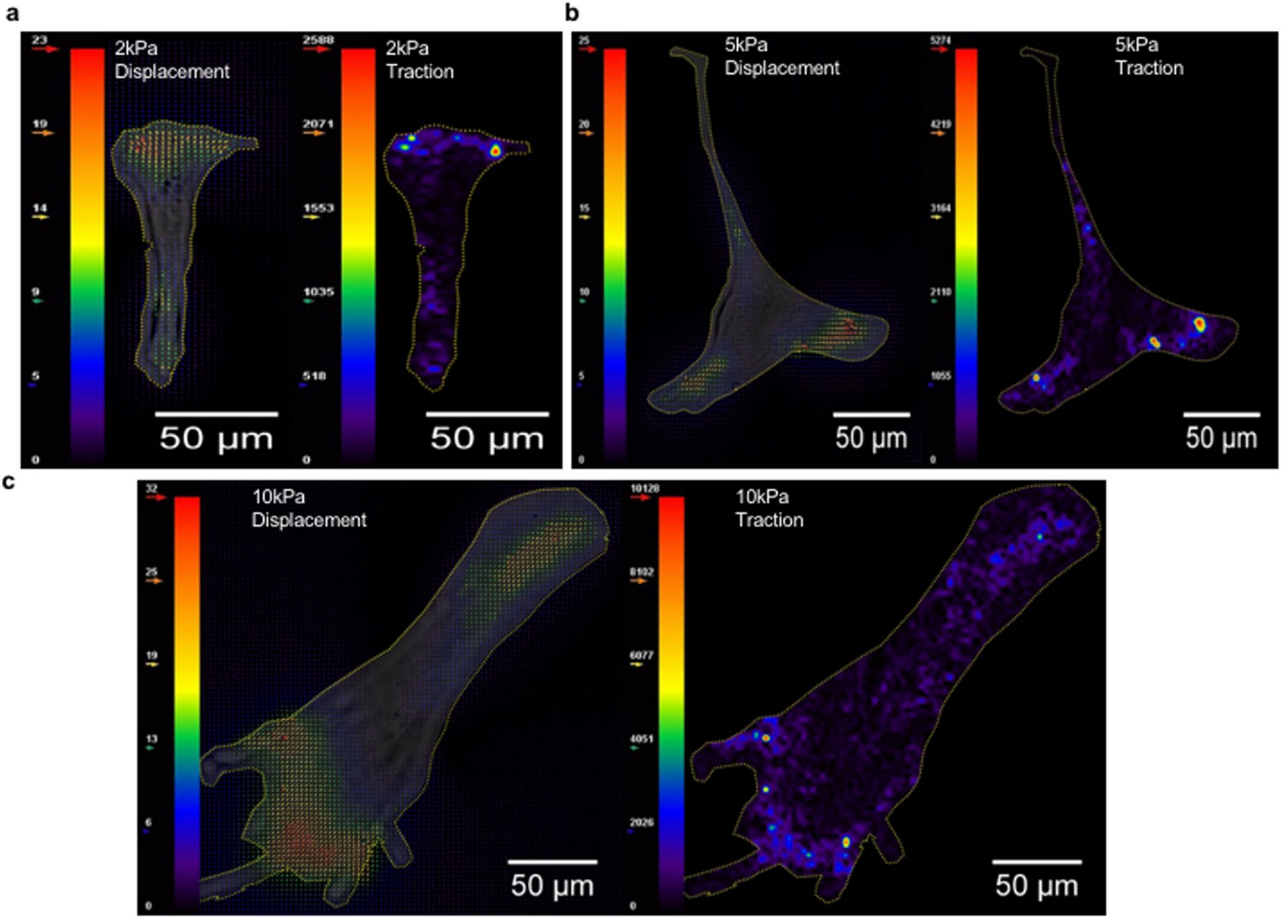
Displacement field and traction map of CCD18 cells on substrates of varying stiffness. (**a–c)** Presents Traction Force Microscopy (TFM) results with CCD18 cells on 2, 5 and 40 kPa substrates respectively. Each image shows substrate displacement vectors and traction stress maps. Cell boundaries are outlined in yellow. Cell spreading area and traction stress increases with increasing stiffness. As a result, total force also increases. However, average substrate deformation remains close for all the substrates. The color scale varies from smaller values for violet to higher values for red. Unit for displacement is pixels (1 pixel = 167 nm) and unit for traction map is Pa.

We induced a CAF phenotype in CRC CCD18 stromal cells by incubation with TGF-β and plated the cells on 2, 10, 40, 95 and 120 kPA polyacrylamide (PA) substrates functionalized by fibronectin. Two kPA substrates correspond to normal colon tissue stiffness^39^, while 10 and 40 kPA substrate are within the range of CRC tumor stiffness^15^. As a control we included 95 and 120 kPa substrates, which are outside the physiological tumor stiffness range. Conditioned media was collected after 72 hours and activin A concentration was measured. We observed that with increasing substrate stiffness, the activin A concentration in the stromal cell conditioned media increased and plateaued at 40 kPa (Fig. 1a). Non-TGF-β transformed quiescent CCD18 colon fibroblasts seeded on the substrates did not secrete significantly increased levels of activin A with increasing substrate stiffness (Supplementary Fig. S1a) To better understand the mechano-biological interplay, we assessed total cell force, substrate deformation, and cell spreading area of CCD18 cells and found that total cell force and cell spreading area increase linearly with higher stiffness while substrate deformation remains constant as shown in Figs. 2 and S1. Vector displacement field for substrate deformation and stress map of representative cells (Fig. 2) show possible biomechanical links. The cell boundaries are outlined, and the images of the cells were overlaid on the displacement plots. It is evident from the images that with increasing substrate stiffness (2, 5 and 10 kPa), cell spreading area and stress magnitude increase.

Next, we investigated activin A’s functional impact on the CAF-induced promigratory phenotype in CRC epithelial cells. Conditioned media from the CCD18 fibroblasts activated by TGF-β cultured on substrates with increasing stiffness was collected and used to treat CRC epithelial FET cells as a model of the impact of stroma on tumor epithelial cells. We assayed cell migration and observed significantly higher cell motility in CRC cells treated with conditioned media from activated CCD18 cells cultured on 10, 40, and 95 kPA substrates compared to the 2 and 120 kPA substrates (Fig. 1b). Migration was highest with the conditioned media from the 40 kPa substrate (Fig. 1c). This correlated to the highest activin A concentration in the conditioned media (Fig. 1a). Furthermore, the addition of follistatin, the ligand trap specific for activin A, reduced CRC cell migration, highlighting the key role of activin A in the induction of cell migration among factors secreted by the stroma (Fig. 1b,c).

### Integrins play an essential role in stromal cell interactions with the ECM

To test whether integrin signaling is needed for activin A secretion, we plated the CCD18 stromal cells on the substrate and added the RGDS peptide to disrupt the integrin-ligand binding and signaling either with or without the addition of TGF-β to activate the stromal cells (Fig. 1d). We observed that inhibiting integrin signaling in stromal cells decreased the amount of secreted activin A indicating that the integrin pathway promotes activin A secretion. RGDS also decreased the secretion of activin A in the presence of TGF-β, albeit not at significant levels.

### Activin A secreted from activated stromal cells promotes EMT

**Figure 3:**
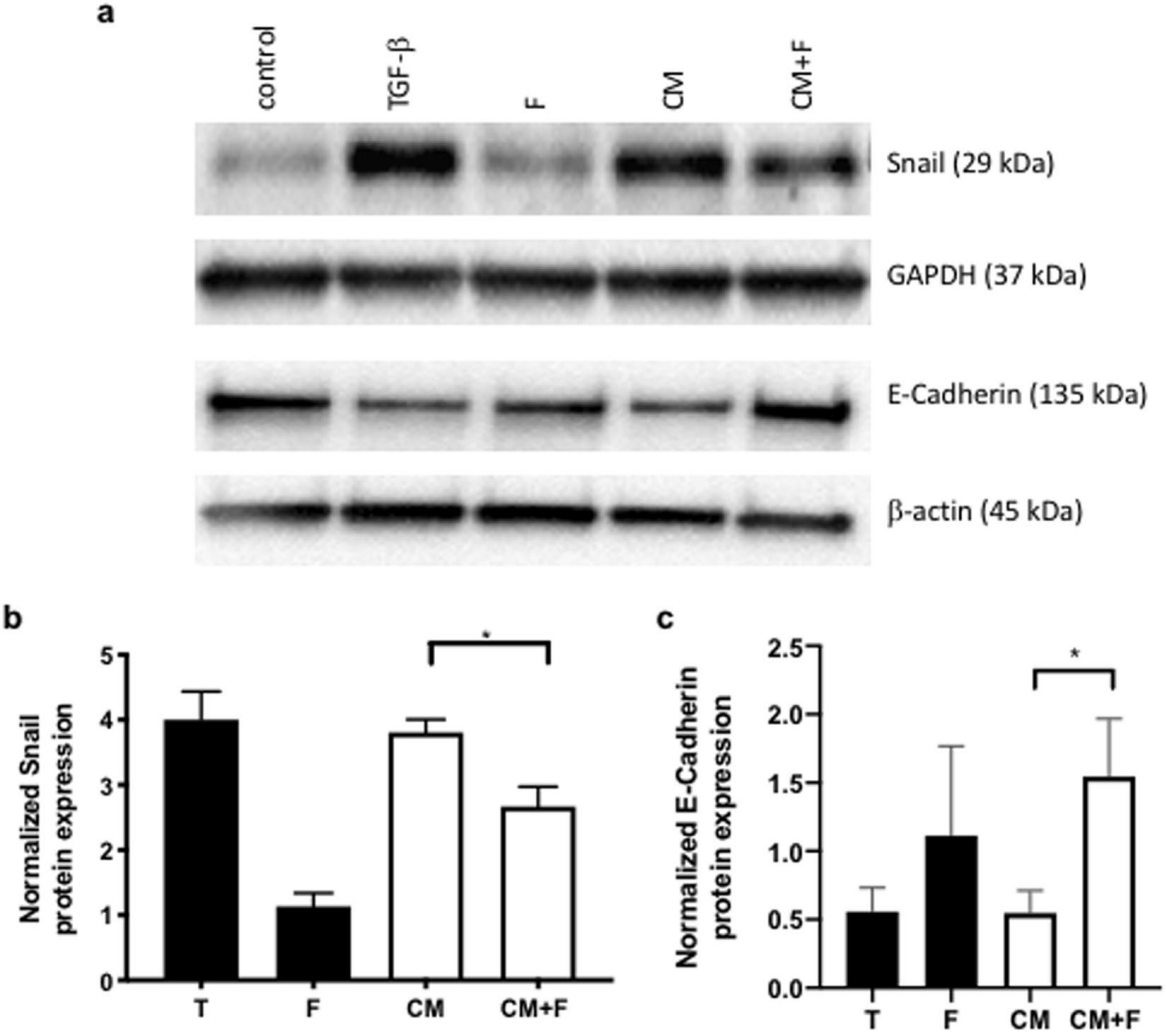
Immunoblot of changes in Snail and E-Cadherin expression. TGF-β and stromal cell conditioned media leads to an increase in EMT-associated activin A signaling in stromal and epithelial cells. (**b**) Densitometry of 3 independent immunoblot replicates normalized to the GAPDH control. Immunoblot of Snail expression in CRC cells (FET) following treatment with TGF-β, follistatin (F), conditioned media (CM) from CCD18 stromal cells, or Conditioned Media (CM) with follistatin (F). Follistatin (F) treatment was used to demonstrate the influence of activin A specifically on Snail expression. (**c**) Densitometry of 3 independent immunoblot of E-Cadherin protein expression replicates normalized to the beta-actin control. (*p < 0.05).

The TME plays an important role in fostering the development of metastasis^40^, and epithelial to mesenchymal transition (EMT) of cancer cells is a hallmark of metastasis. Therefore, we examined the impact of TGF-β -induced stromal cell-derived activin A on markers of EMT (Fig. 3). Treatment of FET cells with TGF-β or CAF-conditioned media (CM) induces a mesenchymal phenotype, as evidenced by increased Snail-expression (Fig. 3a,b), while TGF-β or CM leads to downregulation of the E-Cadherin expression (Fig. 3a,c). These changes in expression of Snail and E-Cadherin were inhibited by the addition of follistatin to the conditioned media confirming the specific role of stromal-derived activin A in the promotion of EMT-associated protein expression.

### Activin A levels are increased in the serum of CRC patients specifically in stage IV cancers and are predictive for CRC metastasis

**Figure 4:**
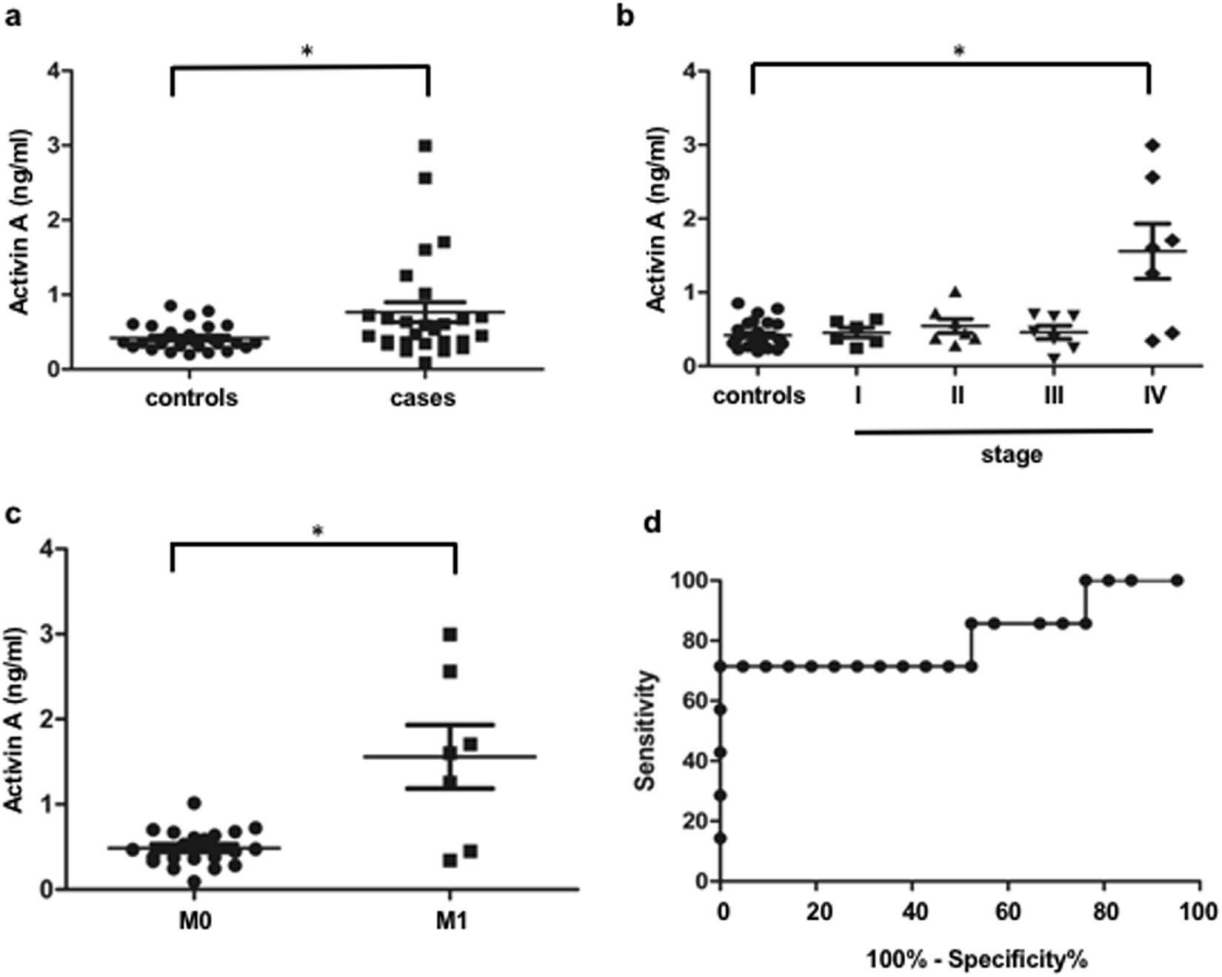
Activin A serum levels are predictive for CRC metastasis. (**a**) ELISA measuring activin A concentration from a cohort of 28 polyp free controls and 28 colon cancer cases. Each stage containing 7 patients. (**b**) A comparison of activin A serum concentration in colon cancer cases stratified by cancer stage. (*p < 0.05). (**c**) comparison of activin A serum concentration in colon cancer patients with metastatic disease (M1) and patients without metastasis (M0). (**d**) ROC analysis to demonstrate the potential of activin A as a predictive indicator for metastasis in CRC. (*p < 0.05).

To determine if our observations of increased activin A in a tissue culture model of CRC is present in CRC patients, we measured activin A levels in serum from a sub-cohort of the Chicago Colorectal Cancer Consortium consisting of 28 control and 28 CRC patients. We matched subject parameters (age, gender, and body mass index) of the control and CRC cases in our cohort to ensure comparability in our study (Table 1). As expected, there was no statistically significant difference between CRC cases and control in parameters of race, age, or sex (Table 1). We measured serum activin A levels in all subjects and observed a higher median activin A level when comparing CRC cases to controls (0.7647 ng/ml versus 0.4179 ng/ml, p = 0.0182, Fig. 4a), which reached statistical significance despite few CRC patients with obvious increased activin A levels. Next, we subdivided our CRC cohort by tumor stages determined by the American Joint Committee on Cancer based on Tumor size, lymph Node affected Metastases (AJCC TNM system) into Union for International Cancer Control (UICC) stages (7 subjects each stage). We compared activin A levels between controls and CRC cases from each stage. We observed a statistically significant 4-fold increase in systemic activin A levels exclusively in late stage metastatic cancers (stage IV) when compared to controls (Fig. 4b). In contrast, activin A levels were not statistically different between controls and cases from pre-metastatic stages I-III.

**Table 1:** Demographic comparison of controls and cases.

Characteristic	Cases (n = 28)	Controls (n = 28)	p-value
**Age:**			
Median	67	66	0.388
Range	(46–83)	(54–81)	
Female sex (%)	12/28 (42.9%)	11/28 (39.3%)	1.000
**Race:**			
Caucasian	17/28	17/28	1.000
African-American	3/28	3/28	1.000
Hispanic	3/28	3/28	1.000
Asian	5/28	5/28	1.000

Given the well-documented pro-metastatic function of activin A in CRC^30^ and the specific increase of activin A in stage IV disease, we investigated activin A levels in CRC patients with metastasis compared to CRC patients without metastases. Serum levels of activin A in metastatic disease (M1) were significantly higher than in patients without organ metastasis (M0) (1.558 ng/ml versus 0.4863 ng/ml, p = 0.0295, Fig. 4c). To investigate activin A’s potential role as an indicator for metastasis in CRC, we performed ROC analysis (Fig. 4d). Activin A serum levels proved to be a good indicator of metastatic disease, with an AUC of 0.8163, with a cutoff of 1.134 ng/ml resulting in a 71.43% sensitivity and 100% specificity.

**Figure 5:**
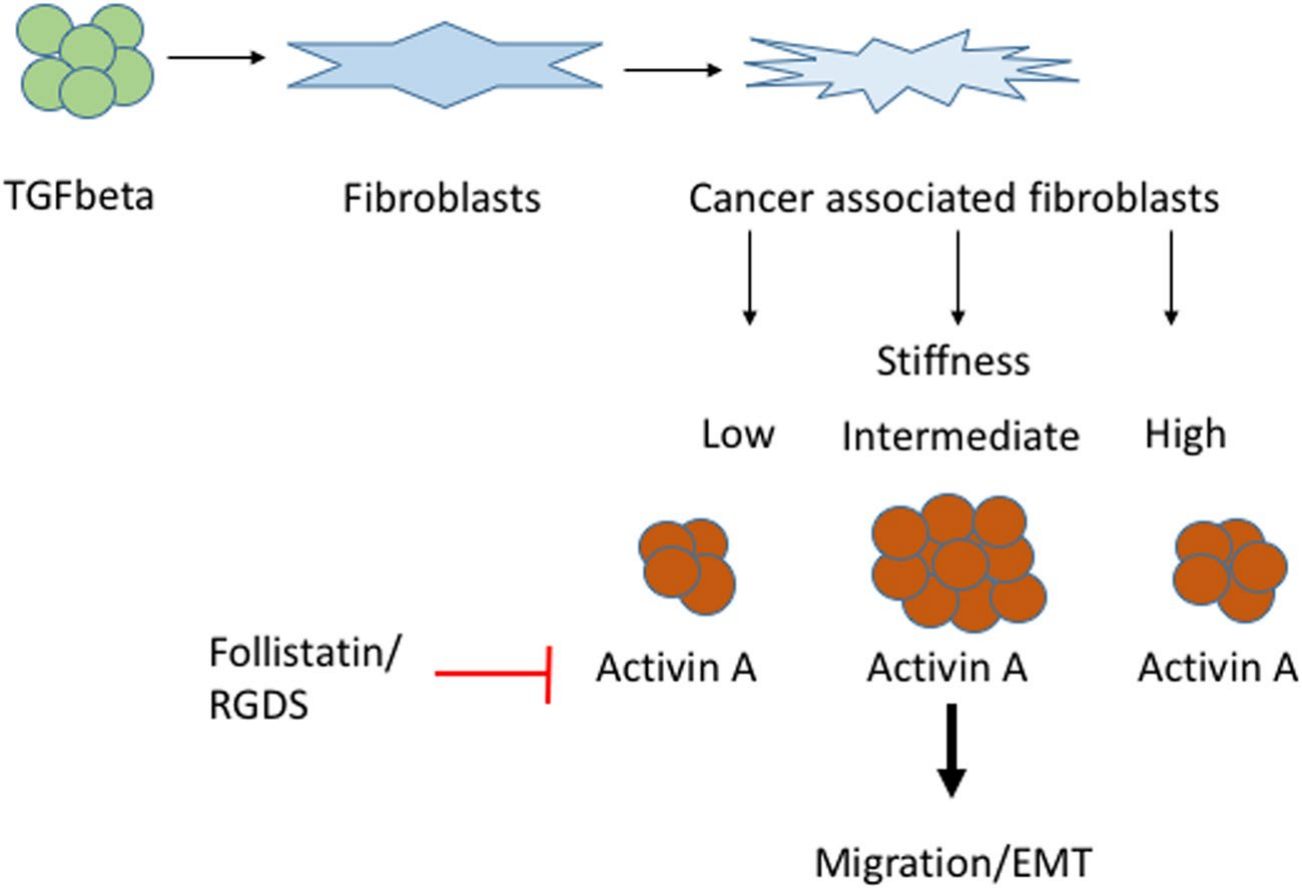
Cancer associated fibroblasts release activin A cultured on intermediate stiffness and leads to cell migration and EMT. TGF-β activates colon stromal fibroblast into cancer associated fibroblasts which secrete activin A. This secretion is enhanced through increased stromal stiffness. The highest level of activin A is released in an intermediate stiffness environment. The increase in activin A can induce migration in colon epithelial cells and lead to EMT resulting in a more metastatic phenotype. Follistatin can counteract this effect. Also the integrin-ligand inhibited through RGDS requires activin A.

In summary, TGF-β activates colon stromal fibroblasts to secrete activin A, and this secretion is enhanced by increased stromal stiffness. The increase in activin A can in turn stimulate colon epithelial cells to migrate and undergo EMT, developing a more metastatic phenotype. These actions require integrin-ligand binding and are specific for activin A as indicated by its inhibition with follistatin (Fig. 5). Elevated serum activin A in CRC patients has the potential to be developed into a marker for metastatic disease allowing for improved therapeutic strategies.

## Discussion

Cancer is lethal due to the complex and poorly understood process of metastasis^41^. From a biophysical standpoint, it has been observed that stiffness of primary tumors is considerably higher than that of normal tissue^42,43^, and this stiffness correlates with cancer progression and metastasis^44,45^. The gradual increase of TME stiffness is attributed to deposition and remodeling of the ECM^44,46–48^ by the stromal environment. Cancer cells, CAFs, and macrophages all work in concert to modulate the ECM within the TME^47^. Biophysical tumor-specific forces in the TME can also lead to the secretion of several cytokines that then influence their surroundings^49^.

TGF-β is a dominant cytokine in colorectal cancers (CRC) and dysregulation of this key signaling pathway contributes to metastatic progression^50^. Increased tissue stiffness leads to conversion of latent TGF-β to activated TGF-β^19,20,22–24^, which results in TGF-β induced responses in asthma^51^ and breast cancer models^38^. We have previously shown that the TGF-β family member activin A is important in CRC metastasis and have recently provided evidence that TGF-β -mediated effects may be via activin A signaling, specifically that TGF-β leads to activin A secretion in intestinal stromal cells^30^.

Our data suggest that TGF-β stimulates activin A secretion from the CAFs in the TME which in turn stimulates epithelial cells to migrate and undergo EMT. The pro-migratory effect from activin A increases with increased force in the tumor microenvironment. Inhibition of activin A with follistatin ameliorates the increased migration indicating that inhibiting activin A and TGF-β pathways could be promising targets for prevention of metastasis.

Our data also reveals, for the first time, that activin A secretion by CAFs increases with substrate stiffness, reaches a peak at stiffness of 40 kPa, and plateaus despite further increases in stiffness. Similarly, CRC epithelial cell migration also peaks with 40 kPa condition media. However, CAF force does not decrease with stiffness, but rather reaches a plateau. In order to explain this observation of stiffness-dependent activin A secretion, we propose a simple biophysical model (Fig. S2). It correlates activin A secretion with the energy spent by the cells to deform the substrate, i.e., cell force times the deformation of the substrate. At lower substrate stiffness, CAF force increases with stiffness (Fig. S1b) maintaining constant deformation (Fig. S1c). Since cell force reaches a plateau beyond a critical stiffness (~20–40 kPa,^52–54^), the cells cannot deform the substrate as stiffness increases, and the work done by the CAFs decreases. Our model captures the experimental trend of stiffness-dependent activin A secretion by CCD18 and suggests a possible mechanistic link between them (Fig. S2). The model predicts a peak in activin A release at a stiffness of 1.25 *K*_0_. Therefore, if *K*_0_ can be determined for the stromal cells collected from patient’s tumor biopsy sample, it might be possible to predict the tumor stiffness at which activin A activity will maximize offering a niche for cancer cells migration.

When assessing serum activin A levels in CRC cases, interestingly, we found that activin A is highly upregulated in stage IV patient samples (Fig. 4) and when tumor stiffness is in the range of 5.58–68 kPa which is significantly higher than those of preceding stages^15,55^. The relatively small subject number per stage is a limitation. A prospective follow-up study is underway to accrue additional samples. Furthermore, we did not have survival data in our cohort, and therefore could not query whether higher activin A levels are predictive for worse prognosis, as it is in lung and pancreatic adenocarcinoma^56,57^. The strong correlation of activin A with metastatic disease suggests that activin A levels may be predictive of outcome in CRC, but confirmation in future studies is needed. Previous studies by others have investigated activin A levels in CRC^58^. On a tissue level, activin A was reported to be strongly upregulated in stage IV CRC^59^, a finding that is in agreement with our observations of increased serum levels in stage IV patients. We previously reported that in the serum from a population of stage II CRC patients from the Quasar 1 study^60^, an increase in the combined score of activin A and TGF-β ligand expression correlates with poor prognosis^30^. Wu *et al*.^58^ report that activin A serum levels can be used to distinguish colorectal cancer patients from healthy controls and patients with polyps in their Chinese cohort; although, they did not have any patients with metastasis in their cohort.

High activin A levels in metastatic disease could imply that activin A plays a functional role in the establishment of metastasis, either through direct action on the tumor epithelial cells or through changes in the microenvironment of the target organs. We found that the high activin A levels in the conditioned media lead to increased expression of Snail, a protein associated with EMT leading to a more metastatic phenotype. Therefore, activin A inhibition in CRC may be an attractive therapeutic target. An important caveat is activin A’s anti-proliferative effects in early disease necessitating the identification of patient subpopulations most suited for this treatment. In addition to potential direct anti-metastatic effects on the tumor, inhibition of activin A has been reported to have a positive impact both on cancer cachexia^57^ and on cancer-associated anemia^61^, two severe aspects of late stage cancers. Overall, activin A inhibition has the potential for a pleiotropic positive effect in late stage CRC patients.

Here, we present data connecting the biophysical TME properties with functionally relevant activin A secretion to affect metastasis. Cells sense various physical cues from their microenvironment and ECM, and transduce them into biochemical signals^62–65^. For instance, they generate cytoskeletal force in response to ECM stiffness. These intracellular forces can expose cryptic sites of signaling molecules, strain intracellular structures to modulate receptor-ligand interactions, up- or down-regulate enzymatic functions, or open mechanosensitive ion channels^65^. Hence, we anticipated a possible link between substrate deformation and traction force and activin A pathway. Our experiments suggest that CCD18 fibroblasts sense substrate stiffness and regulate activin A secretion, which is at least partially dependent on integrin signaling. However, the fibroblasts maintain a constant substrate deformation (~700–900 nm) independent of stiffness in the range of 2 to 10 kPa by increasing force proportionately (Fig. S1b,c). Yip *et al*.^66^ also reported stiffness independent substrate deformation (400 nm) by NIH3T3 fibroblasts cultured on PA gel substrate of stiffness below 20 kPa. Cell spreading area also increases linearly up to substrate stiffness of 10 kPa (Fig. S1d). However, our experiment shows that activin A production by CCD18 cells has a baseline value independent of substrate stiffness, suggesting a stiffness-independent component. Activin A secretion increases with stiffness, reaches a maximum, and then decreases, in contrast to the contractile force that increase monotonically with stiffness and reaching a plateau. This implies that (a) a competing retardation mechanism exists against activin A secretion that may come into play with increasing stiffness, and (b) the mechano-signaling role of transmembrane proteins in activin A production may not be critical, as they would remain stretched with increasing cell force and stiffness.

In conclusion, we suggest that increased tumor microenvironment stiffness leads to stromal cell magnification of the pro-oncogenic function of TGF-β through thus far unrecognized induction and utilization of activin A signaling that may represent a potential therapeutic target in advanced stage CRC.

## Material and Methods

### Colon cancer cell lines

CCD18 fibroblasts (ATCC, Manassas, VA, USA) were maintained in Dulbecco’s Modified Eagles media, and FET cells (gift from Michael Brattain, University of Nebraska, Omaha, NE, USA) were maintained in Dulbecco’s Modified Eagles medium/F12 50:50 (both Invitrogen, Carlsbad, CA, USA) supplemented with 10% fetal bovine serum and penicillin (100 U/ml)/streptomycin (100 µg/ml) (Invitrogen). Cells were grown at 37 °C in a humidified incubator with 5% CO_2_. All cells were serum starved for 24 hours prior to treatment to approximate cell cycle synchronization. Cells were validated by 9 STR (short tandem repeat) profiling using CellCheck 9 Plus and tested for mycoplasma (both IDEXX, Columbia, MO, USA).

### Reagents and antibodies

Activin A and TGFβ1 were reconstituted in 4 mM HCl according to the manufacturer’s instruction (both R&D, Minneapolis, MN, USA). Final concentrations used were each 10 ng/ml, respectively. Human recombinant follistatin 288 (R&D, Minneapolis, MN, USA) was reconstituted in PBS and used at a final concentration of 100 ng/ml. RGDS peptide (bio-techne, Minneapolis, MN, USA) was reconstituted in 1 mg/ml water and used at a final concentration of 10 ng/ml. For Western blotting we used antibodies against Snail, E-Cadherin, and GAPDH (all from Cell Signaling Technology, Danvers, MA, USA).

### Quantification of serum activin A

Activin A from all samples was measured utilizing the Activin A Quantikine colorometric sandwich Enzyme Linked Immunosorbent Assay (ELISA) following the manufacturer’s instructions (R&D systems, Minneapolis, MN). All patient samples were run in duplicates after a 1:4 dilution in PBS (Corning Inc., Corning, NY). Supernatant from CCD18 fibroblasts were diluted 1:1. In addition to the samples from this study, samples spiked with human recombinant Activin A (R&D systems) were included to ensure assay validity.

### Polyacrylamide substrates

A protocol by Tse and Engler^67^ was closely followed for synthesis of Polyacrylamide (PA) gels. Clean glass coverslips (No. 1, Ted Pella) were silanized with (3-Aminopropyl)trimethoxysilane (APTES) solution (Sigma-Aldrich) and then functionalized with 0.5% glutaraldehyde solution (Polysciences). Acrylamide (40% sol., Sigma-Aldrich), N,N′-Methylenebis- acrylamide (2% sol., Sigma-Aldrich) and Dulbecco’s Phosphate Buffered Saline (DPBS, Corning) were mixed at different ratios according to the protocol for various Young’s moduli (2, 10 and 40 kPa). 1% (v/v) of ammonium persulfate (APS) solution (10% w/v APS, Bio-Rad) and 0.1% (v/v) of Tetramethylethylenediamine (TEMED) solution (Bio-Rad) was used to catalyze the polymerization reaction. After transfer of the coverslips with PA gel to 24-well plates, 0.2 mg/ml sufosuccinimidyl-6- (4′-azido- 2′-nitrophenylamino)- hexanoate (Sulfo-SANPAH, Thermo Scientific) solution in HEPES buffer (50 mM HEPES at pH 8.5, Fisher Scientific) was applied and UV activated. For protein functionalization, the substrates were then immersed overnight in 25 µg/ml Fibronectin (Human, Corning) solution in HEPES buffer. The substrates were then rinsed 3 times with DPBS and standard procedure was followed for subsequent cell culture studies. To estimate the stiffness of the gels, reported values by Tse and Engler were considered. The elastic moduli were measured using atomic force microscopy (AFM) which is a nano-indentation method of calculating elasticity. We verified a few specimens using micro-indentation techniques developed in our lab and the results match the published values.

### Traction force microscopy (TFM)

In order to investigate the mechanical response of the fibroblasts to increasing matrix stiffness, traction force microscopy experiments were performed on PA gel substrates embedded with 100 nm red beads (excitation/emission −580/605 nm, Thermo-Fisher) as fiducial markers. The detailed method of substrate preparation is described by Knoll *et al*.^68^. Fluorescent images of the beads were taken with and without the cells on the substrates. These images were compared and analyzed for displacements/deformations and stresses generated by the cells. Traction stress/force was quantified using the following equations: maximum traction stress = (${({\tau }_{x}^{2}+{\tau }_{y}^{2})}^{1/2}$) and total traction force = ($\,{\sum }_{cellarea}|{({\tau }_{x}^{2}+{\tau }_{y}^{2})}^{1/2}\,dA|\,$), where $\tau$ is local traction stress around area dA^69,70^. Analysis of TFM images was performed using ImageJ open-source software.

### Conditioned media and migration assay

Before we performed migration assay, conditioned media from human stromal colon cells (CCD18) was generated by seeding CCD18 cells on substrates with increasing stiffness (2 kPa, 10 kPa, 40 kPa, 95 kPa and 120 kPa). After 24 hours of serum starvation, the cells were treated for 72 hours with 10 ng/ml TGF-β to generate conditioned media. This media was used on the epithelial cells and cell migration was measured as previously described^28,30^. Briefly, transwell 12 well plates (8 µm pores, Corning, NY, USA) coated with fibronectin (Sigma, St. Louis, MO, USA) were seeded with 5 × 10^5^ colon cancer cells per well. Cells were allowed to migrate for 6 hours, stained with DAPI, and imaged. Images from 5 microscopic fields at the center of each well were counted using ImageJ32 software (NIH). We used automated counting of single-color images in ImageJ32 to quantify the migrated cells.

### EMT assay and western blotting

Stromal cells (CCD18) were treated with TGF-β with or without 30 min prior treatment with follistatin for 72 hours to generate conditional media. This media was applied to FET epithelial cells to induce EMT. After 96 hours, epithelial FET cells were lysed using CHAPS lysis buffer (containing 20 mM Bicine pH 7.6 and 0.6% Chaps) with added protease and phosphatase inhibitors. Western blots were performed as previously described^28,30^. Briefly, Western blotting was performed using standard protocols with 4–20% gradient polyacrylamide gels, transferred to a nitrocellulose membrane, overnight incubation with a primary antibody followed by horseradish peroxidase-linked secondary antibody incubation and ECL detection. Visualization and quantification of chemiluminescence was done with ChemiDoc MP from BioRad. As a marker for epithelial cells E-Cadherin was used and for mesenchymal cells Snail.

### Patient cohort

Informed consent was received from all human subjects, and no subject was under the age of 18. All methods were carried out in accordance with relevant guidelines and regulations of the University of Illinois at Chicago, and all experimental protocols were approved by the Institutional Review Board of the University of Illinois at Chicago. The subjects were part of the Chicago Colorectal Cancer Consortium (CCCC) cohort, a multi-center effort to increase understanding of pathogenesis of and racial disparities in CRC. Seven subjects from each stage (I-IV), plus twenty-eight controls matched for age, race and gender were included in this study, for a total of 56 individuals in the study. All controls had no current GI symptoms; a screening colonoscopy in the last 3 months prior to inclusion; and were polyp free at the time study inclusion. Diagnosis and staging of cases was done following the American Joint Committee on Cancer (AJCC) guidelines. Serum samples were aliquoted after blood draw and stored at −80 °C. Repeat freeze/thaw cycles were avoided.

### Statistical analysis

For Comparison of means, two-sided t-test with Welch’s correction was used when two groups were compared, and one-way Analysis of Variance (ANOVA) with Dunett’s post-test was used for comparison of three or more groups. For statistical purposes, gender and race was considered a binary variable, even though we acknowledge limitations of this approach. Fisher’s exact test was used for analysis of distribution of binary variables in cases and control groups. In order to assess serum activin A as an indicator of metastasis in CRC, Receiver Operator Characteristics (ROC) analysis was performed, and the Area Under the Curve (AUC) was calculated. All statistical analysis was performed using GraphPad Prism version 5.00 for Windows (GraphPad Software, San Diego, CA). Differences between two groups were determined using the Student’s *t*-test. Probability values less than 0.05 were considered significant. Biological replicates from 3–5 experiments represent the data shown.

## Supplementary information

Supplementary Information

Supplementary Information 2

## Data Availability

All data generated or analyzed during this study are included in this published article (and its Supplementary Information files).
