# Author Correction: Increased stiffness of the tumor microenvironment in colon cancer stimulates cancer associated fibroblast-mediated prometastatic activin A signaling

**DOI:** 10.1038/s41598-020-64239-2

**Published:** 2020-04-30

**Authors:** Jessica Bauer, Md Abul Bashar Emon, Jonas J. Staudacher, Alexandra L. Thomas, Jasmin Zessner-Spitzenberg, Georgina Mancinelli, Nancy Krett, M. Taher Saif, Barbara Jung

**Affiliations:** 1 Department of Medicine, Division of Gastroenterology and Hepatology, University of Illinois at Chicago, Chicago, IL USA; 2 Department of Mechanical Science and Engineering, University of Illinois at Urbana-Champaign, Urbana, IL USA; 3 Department of Gastroenterology, Infectious Diseases and Rheumatology, Charité-University Medicine, Berlin, Germany; 4 Berlin Institute of Health (BIH), Berlin, Germany; 5 Medical University of Vienna, Vienna, Austria

Correction to: *Scientific Reports* 10.1038/s41598-019-55687-6, published online 09 January 2020

The original version of this Article contained a typographical error in the spelling of the author Md Abul Bashar Emon, which was incorrectly given as Md Abdul Bashar Emon. This has now been corrected in the PDF and HTML versions of the Article, and in the accompanying Supplementary Information files.

