# Supplementary material for: Increased stiffness of the tumor microenvironment in colon cancer stimulates cancer associated fibroblast-mediated prometastatic activin A signaling
Source: Sci Rep. 2020 Jan 9;10:50. doi: 10.1038/s41598-019-55687-6 (PMC6952350; doi:10.1038/s41598-019-55687-6)
Supplement: Supplementary file 1 — Supplementary Information [file 41598_2019_55687_MOESM1_ESM.pdf]

Supplementary Data

Title: Increased stiffness of the tumor microenvironment in colon cancer stimulates cancer associated fibroblast-mediated prometastatic activin A signaling

Authors: Jessica Bauer, Md Abdul Bashar Emon, Jonas J Staudacher, Alexandra L Thomas, Jasmin Zessner-Spitzenberg, Georgina Mancinelli, Nancy Krett, M Taher Saif, and Barbara Jung

Biophysical model relating activin secretion by CCD18 and substrate stiffness details:
Cells sense various physical clues from their microenvironment and extracellular matrix (ECM), and transduce them into biochemical signals[1-4]. For instance, they generate cytoskeletal force in response to ECM stiffness. These intracellular forces can expose cryptic sites of signaling molecules, strain intracellular structures to modulate receptor-ligand interactions, up- or down-regulate enzymatic functions, or open mechanosensitive ion channels[4]. On 2D substrates, cells sense substrate rigidity by contractile force and by engaging transmembrane proteins, actin cytoskeleton and various adhesion molecules (e.g. talin, vinculin, filamin, a-actinin, tensin)[5-12]. Cell-force increases with substrate stiffness until a threshold stiffness is reached when force reaches a plateau[5]. Stiffer substrates lead to robust actin structure formation, change in nuclear volume and shape and cellular fate decisions[1-4].

In order to explain our experimental observation, we propose the following model. Consider a cell attached to a compliant 2D substrate with stiffness K_s (Supplementary Figure 2). Its acto-myosin machinery generates contractile force, F, and deforms the substrate by $\delta_s = F/K_s$. The intracellular force is transferred to the substrate through the transmembrane and the ECM molecules with combined stiffness of K_{EC+TM}. Their deformation is $\delta_{ECM+TM} = F/K_{EC+TM}$. With increasing K_s, the cell increases acto-myosin machinery and adhesion sites. Beyond a certain K_s, the rate of increase of F with K_s decreases, and F reaches a plateau. Correspondingly δ_s decreases and eventually vanishes at very high stiffness. We assume that δ_{ECM+TM} is small compared to δ_s for most substrate stiffness. Furthermore, if the strain of F-actin is ignored, then δ_s is equal to the walking of myosin motors or sliding of F-actin filaments within the cell. With increasing K_s, the number of myosin motors increases. Their cumulative number of steps increases with substrate stiffness, but decreases with δ_s when K_s increases beyond threshold. The number of cumulative steps then scales with $F\delta_s$ - the work done by the cell on the elastic substrate. The stiffness dependent part

of activin production seems to follow the cumulative myosin steps or $F\delta_s$ which possibly serves as the intracellular signal. We thus propose that the stiffness dependent component of activin, A_{st}, produced by the cells in a given time is

$$A_{st} \propto F\delta_s$$

Since F increases with K_s and reaches a plateau, F_{peak}, we write $F = F_{peak}(1 - \exp(-\lambda))$, where $\lambda = K_s/K_0$, and K_0 is a constant.

Since $A_{st} \propto F\delta_s$,

we write $A_{st} = A_0 \frac{(1-e^{-\lambda})^2}{\lambda}$, where A_0 is constant.

For fibroblasts, K_0 is estimated to be within 20-40 KPa[13-16]. Figure S4 shows A_{st} for K_0 = 20, 30, 40 KPa, and A_0 = 0.1 ng/ml, together with experimental values (stiffness dependent activin component).

Figure S1

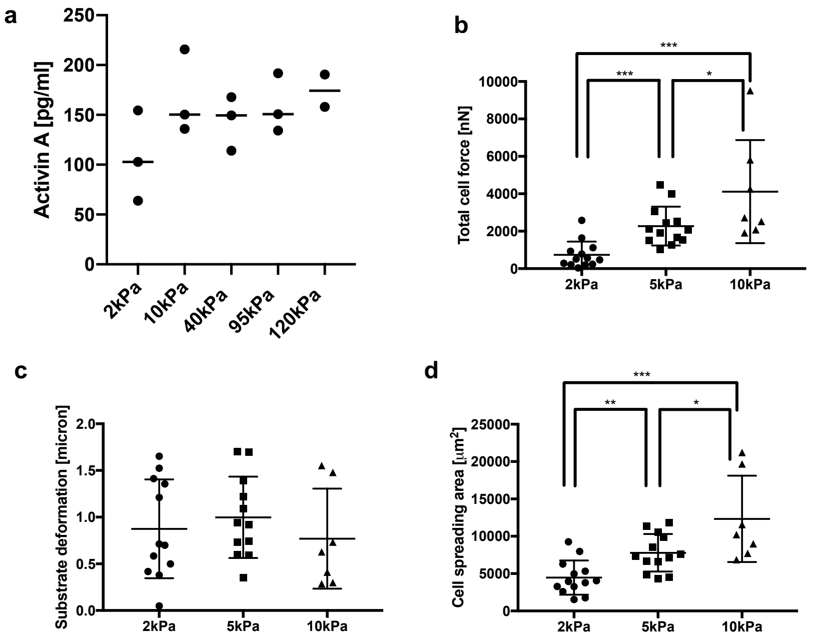

Figure S1: Parameters of substrate force at increasing stiffness. (a) Activin A levels from quiescent colon fibroblasts CCD18 seeded on substrates (**b**) Total traction force as calculated by ($\sum_{cell\ area} |(\tau_x^{\,2} + \tau_y^{\,2})^{1/2}\ dA|$) increases linearly with increasing substrate stiffness up to 10 kPa.

(**c**) Average substrate deformation computed at nodal points of a grid under the cell remains similar despite varying rigidity. (**d**) Cell spreading area also increases linearly with increasing stiffness indicating higher cytoskeletal stretch and nuclear deformation on stiffer substrates.

Figure S2

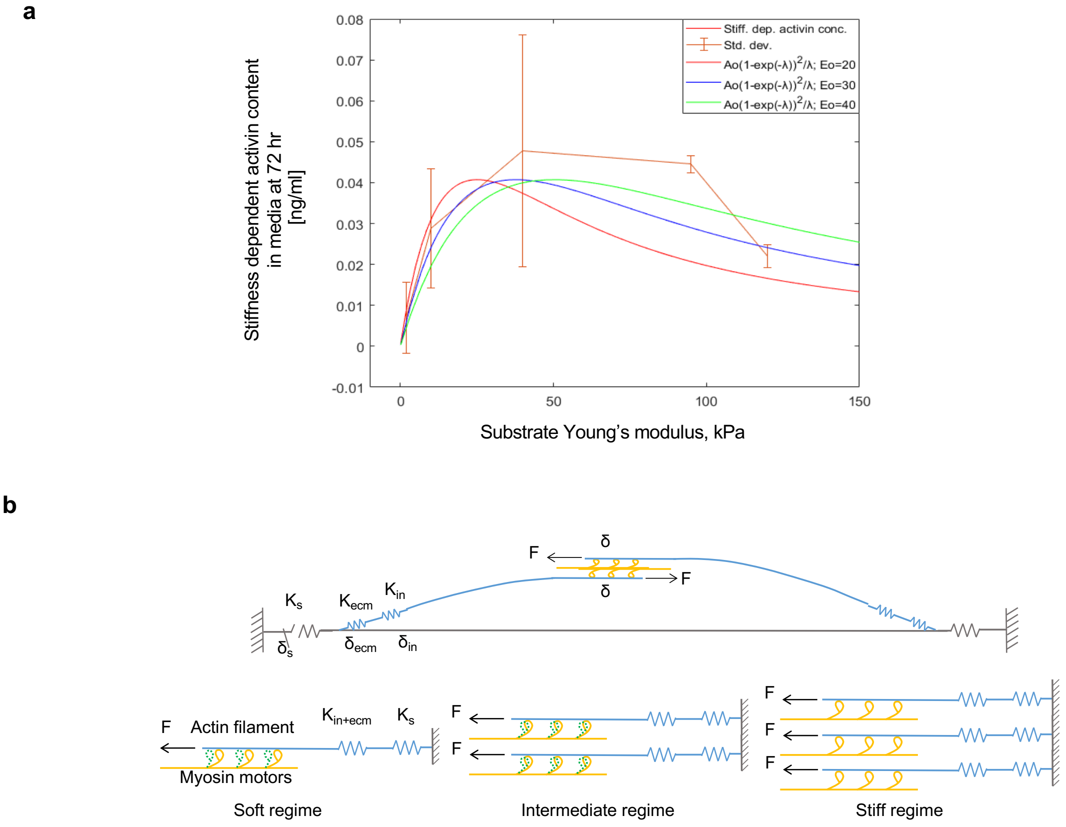

Figure S2: Stiffness-dependent activin release model captures the experimentally obtained trend and Acto-myosin activity on varying substrate stiffness. (a) Models with Eo = 20, 30 and 40 has been presented with the stiffness-dependent activin content in fibroblast culture media after 72 hours. Model with Eo=30 which shows a peak concentration at 37.5 kPa seems to fit best with the data. For other cells lines and primary cells, this parameter can help us predict the stromal stiffness that can most likely to promote metastasis. (b) Within soft regime, as substrate stiffness increases, traction force is increased to produce a certain deformation. In the intermediate regime, traction force is increased by adding acto-myosin filaments, but similar deformation is not produced since the substrate is stiffer and myosin heads cannot complete as many steps as on soft substrate. In the stiff regime, there is almost no deformation although traction force is the highest and thus little or no walking of myosin heads (K_s: substrate stiffness; F: force; K_{ecm}; ECM stiffness; K_{in}: increasing stiffness; delta: change).

Supplementary References for Biophysical Model:

1 Silver, F. H. & Siperko, L. M. Mechanosensing and mechanochemical transduction: how is mechanical energy sensed and converted into chemical energy in an extracellular matrix? *Crit Rev Biomed Eng* **31**, 255-331 (2003).

2 Shemesh, T., Geiger, B., Bershadsky, A. D. & Kozlov, M. M. Focal adhesions as mechanosensors: a physical mechanism. *Proc Natl Acad Sci U S A* **102**, 12383-12388, doi:10.1073/pnas.0500254102 (2005).

3 Vogel, V. Mechanotransduction involving multimodular proteins: converting force into biochemical signals. *Annu Rev Biophys Biomol Struct* **35**, 459-488, doi:10.1146/annurev.biophys.35.040405.102013 (2006).

4 Vogel, V. & Sheetz, M. Local force and geometry sensing regulate cell functions. *Nat Rev Mol Cell Biol* **7**, 265-275, doi:10.1038/nrm1890 (2006).

5 Moore, S. W., Roca-Cusachs, P. & Sheetz, M. P. Stretchy proteins on stretchy substrates: the important elements of integrin-mediated rigidity sensing. *Dev Cell* **19**, 194-206, doi:10.1016/j.devcel.2010.07.018 (2010).

6 Delon, I. & Brown, N. H. Integrins and the actin cytoskeleton. *Curr Opin Cell Biol* **19**, 43-50, doi:10.1016/j.ceb.2006.12.013 (2007).

7 Geiger, B. & Yamada, K. M. Molecular architecture and function of matrix adhesions. *Cold Spring Harb Perspect Biol* **3**, doi:10.1101/cshperspect.a005033 (2011).

8 Choi, C. K. *et al.* Actin and alpha-actinin orchestrate the assembly and maturation of nascent adhesions in a myosin II motor-independent manner. *Nat Cell Biol* **10**, 1039-1050, doi:10.1038/ncb1763 (2008).

9 Burridge, K. & Mangeat, P. An interaction between vinculin and talin. *Nature* **308**, 744-746 (1984).

10 Li, J., Han, D. & Zhao, Y. P. Kinetic behaviour of the cells touching substrate: the interfacial stiffness guides cell spreading. *Sci Rep* **4**, 3910, doi:10.1038/srep03910 (2014).

11 Saez, A., Buguin, A., Silberzan, P. & Ladoux, B. Is the mechanical activity of epithelial cells controlled by deformations or forces? *Biophys J* **89**, L52-54, doi:10.1529/biophysj.105.071217 (2005).

12 Jiang, G., Huang, A. H., Cai, Y., Tanase, M. & Sheetz, M. P. Rigidity sensing at the leading edge through alphavbeta3 integrins and RPTPalpha. *Biophys J* **90**, 1804-1809, doi:10.1529/biophysj.105.072462 (2006).

13 Marion Ghibaudo, A. S., Léa Trichet, Alain Xayaphoummine, Julien Browaeys, Pascal Silberzan, Axel Buguin, Benoît Ladoux. Traction forces and rigidity sensing regulate cell functions. *Soft Matter* **4**, 1839-1843, doi:10.1039/B804103B (2008).

14 Shenoy, V. B., Wang, H. & Wang, X. A chemo-mechanical free-energy-based approach to model durotaxis and extracellular stiffness-dependent contraction and polarization of cells. *Interface Focus* **6**, 20150067, doi:10.1098/rsfs.2015.0067 (2016).

15 Trichet, L. *et al.* Evidence of a large-scale mechanosensing mechanism for cellular adaptation to substrate stiffness. *Proc Natl Acad Sci U S A* **109**, 6933-6938, doi:10.1073/pnas.1117810109 (2012).

16 Mitrossilis, D. *et al.* Single-cell response to stiffness exhibits muscle-like behavior. *Proc Natl Acad Sci U S A* **106**, 18243-18248, doi:10.1073/pnas.0903994106 (2009).
