# Supplementary material for: Increased stiffness of the tumor microenvironment in colon cancer stimulates cancer associated fibroblast-mediated prometastatic activin A signaling
Source: Sci Rep. 2020 Jan 9;10:50. doi: 10.1038/s41598-019-55687-6 (PMC6952350; doi:10.1038/s41598-019-55687-6)
Supplement: Supplementary file 2 — Supplementary Information 2 [file 41598_2019_55687_MOESM2_ESM.pdf]

Supplementary Information

Title: Increased stiffness of the tumor microenvironment in colon cancer stimulates cancer associated fibroblast-mediated prometastatic activin A signaling

Authors: Jessica Bauer, Md Abdul Bashar Emon, Jonas J Staudacher, Alexandra L Thomas, Jasmin Zessner-Spitzenberg, Georgina Mancinelli, Nancy Krett, M Taher Saif, and Barbara Jung

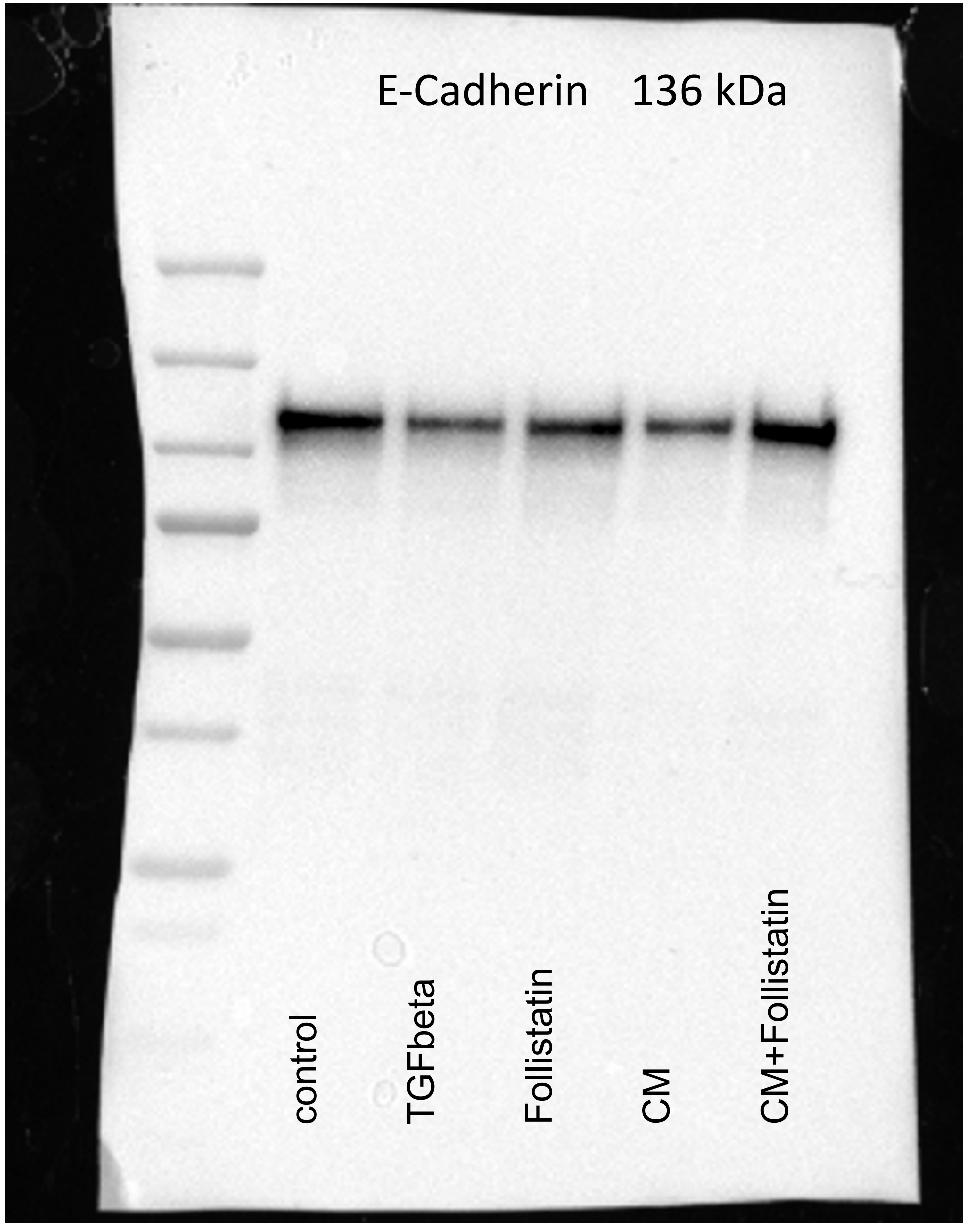

250kDa
150kDa
100kDa
75kDa
50kDa
37kDa
25kDa
20kDa

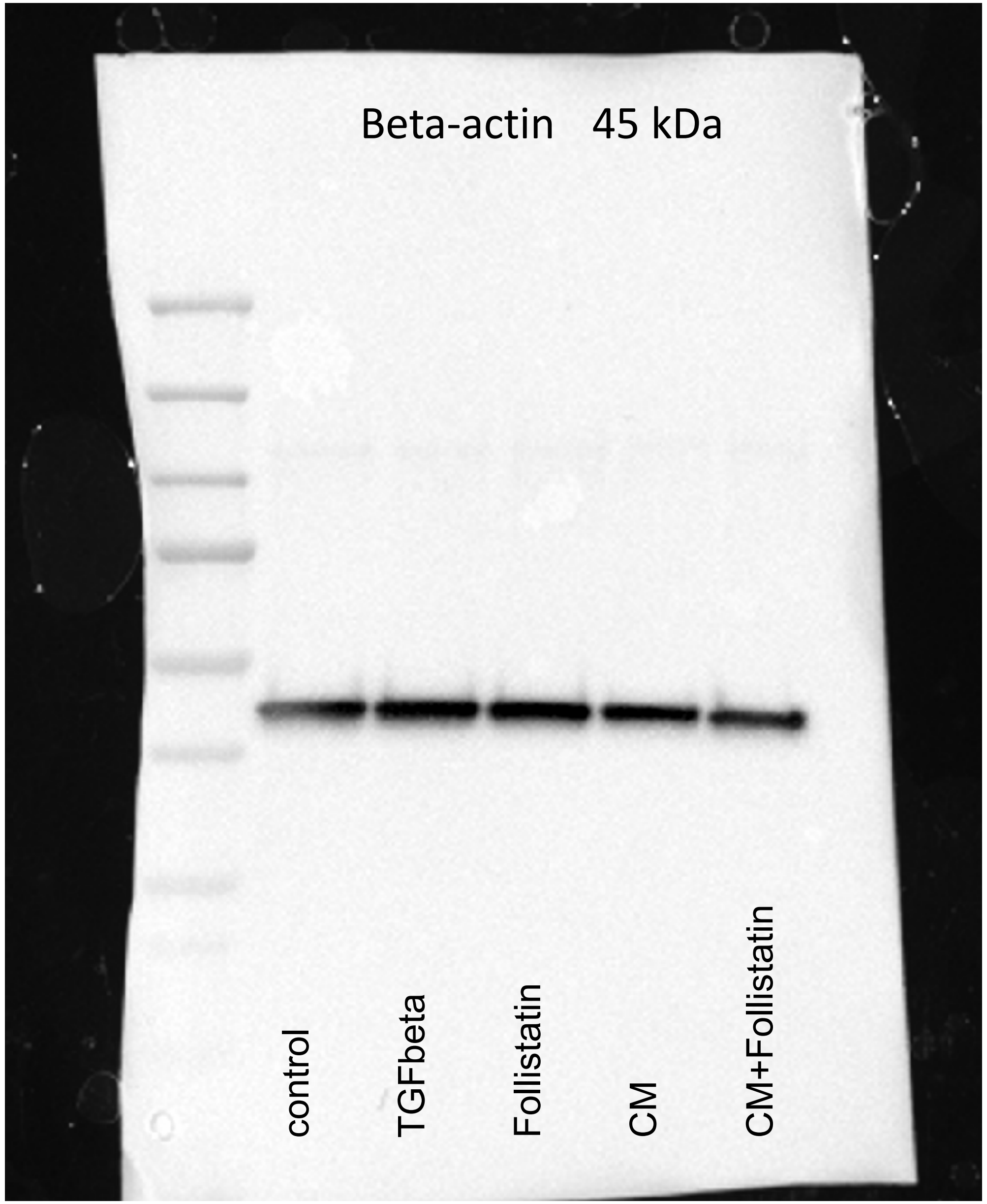

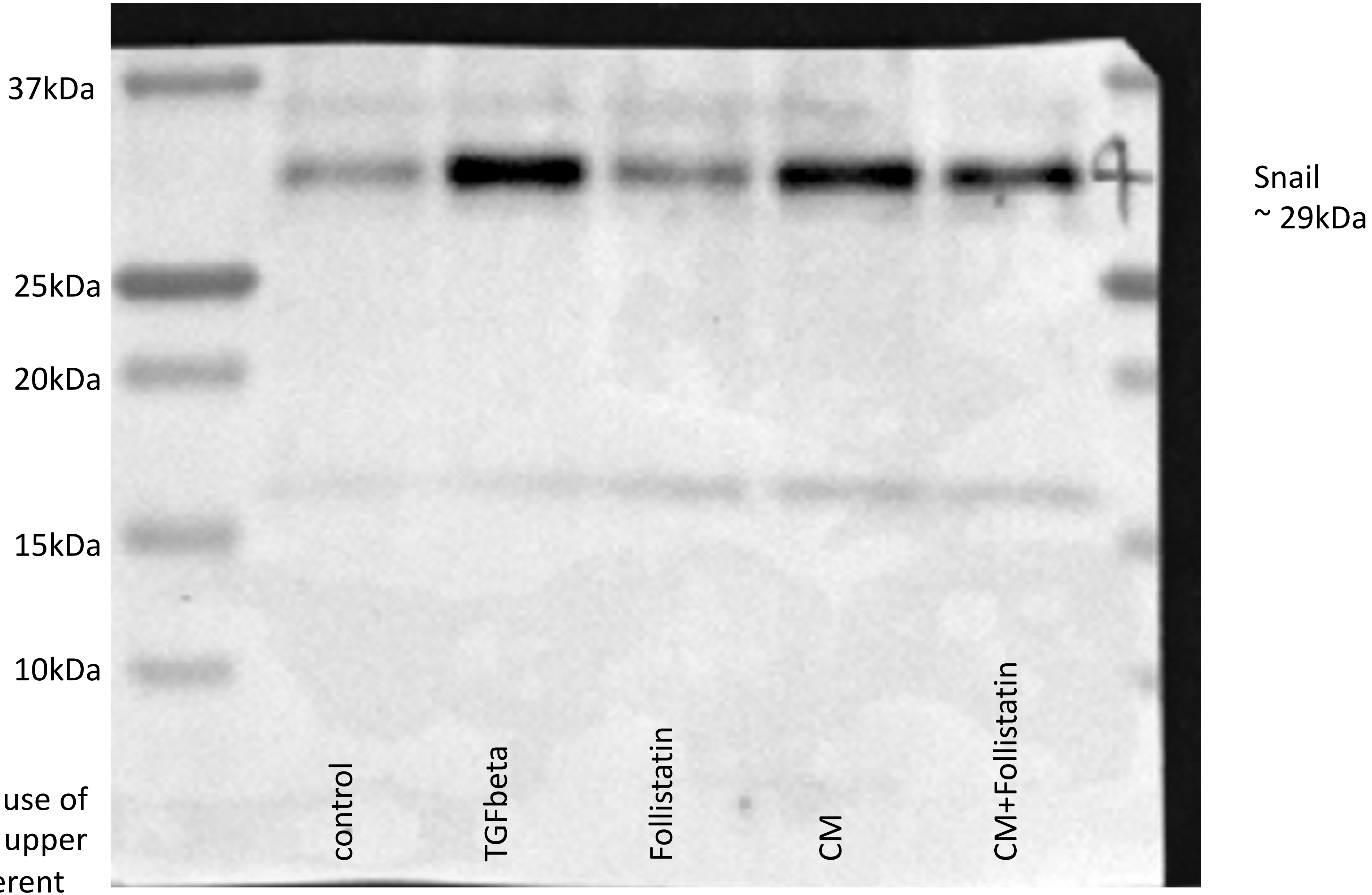

Membrane was cut because of economical reasons and upper part was probed for different antibody

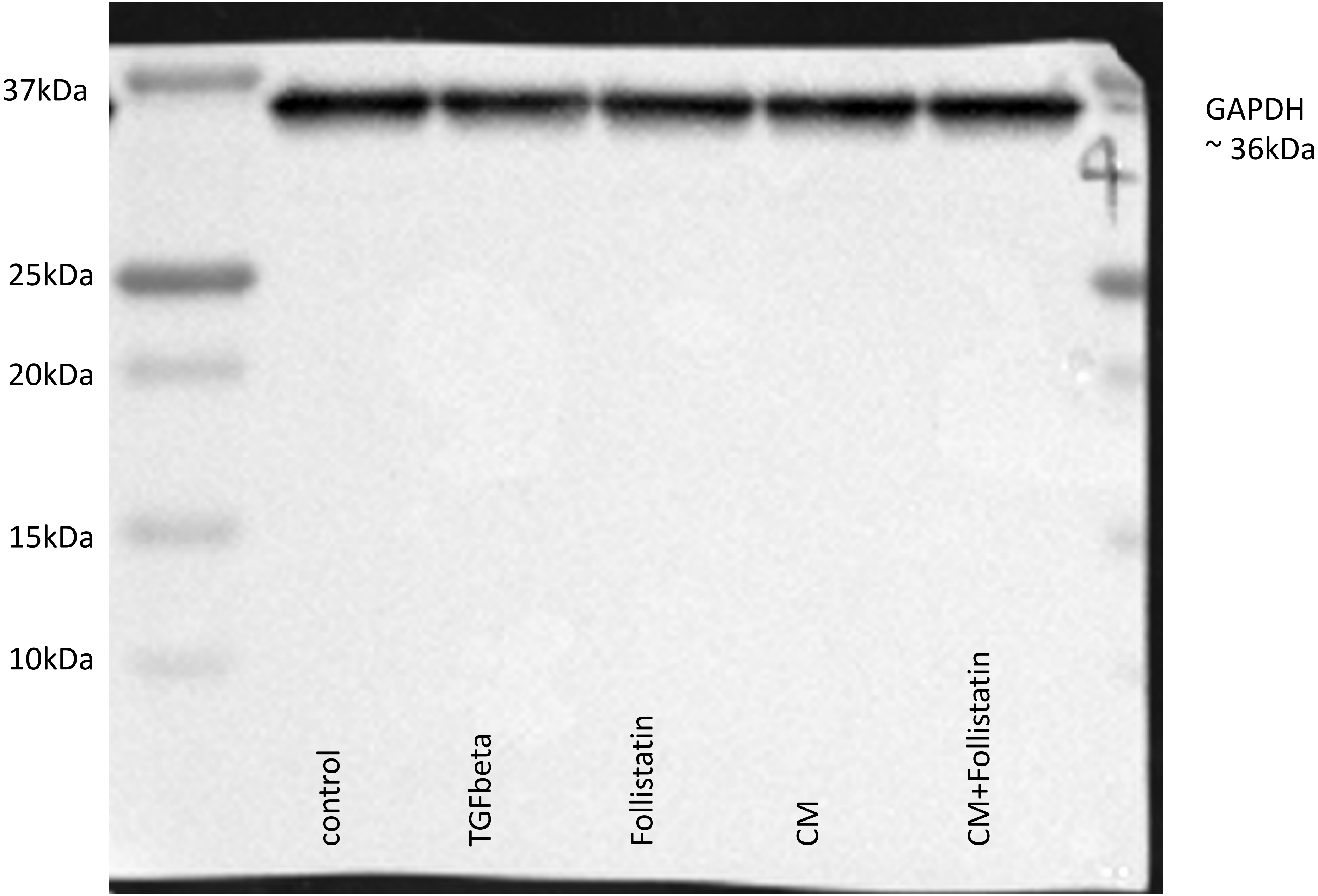
37kDa
25kDa
20kDa
15kDa
10kDa
GAPDH
~ 36kDa
control
TGFbeta
Follistatin
CM
CM+Follistatin
